# Systematic evaluation of genetic mutations in ALS: a population-based study

**DOI:** 10.1136/jnnp-2022-328931

**Published:** 2022-07-27

**Authors:** Maurizio Grassano, Andrea Calvo, Cristina Moglia, Luca Sbaiz, Maura Brunetti, Marco Barberis, Federico Casale, Umberto Manera, Rosario Vasta, Antonio Canosa, Sandra D’Alfonso, Lucia Corrado, Letizia Mazzini, Clifton Dalgard, Ramita Karra, Ruth Chia, Bryan Traynor, Adriano Chiò

**Affiliations:** 1 Department of Neuroscience, University of Turin, Torino, Italy; 2 Neuromuscular Diseases Research Section, Laboratory of Neurogenetics, Porter Neuroscience Research Center, National Institute on Aging, Bethesda, Maryland, USA; 3 S.C. Neurologia 1U, Azienda Ospedaliero Universitaria Citta della Salute e della Scienza di Torino, Torino, Italy; 4 Laboratory of Genetics, Department of Clinical Pathology, Azienda Ospedaliero Universitaria Citta della Salute e della Scienza di Torino, Torino, Italy; 5 Department of Health Sciences, Interdisciplinary Research Center of Autoimmune Diseases, University of Eastern Piedmont Amedeo Avogadro School of Medicine, Novara, Piemonte, Italy; 6 Department of Neurology, University Hospital Maggiore della Carità, Novara, Italy; 7 Department of Anatomy, Physiology & Genetics, Uniformed Services University of the Health Sciences, Bethesda, Maryland, USA; 8 The American Genome Center, Collaborative Health Initiative Research Program, Uniformed Services University of the Health Sciences, Bethesda, Maryland, USA; 9 Department of Neurology and Neurosurgery, Johns Hopkins University, Baltimore, Maryland, USA; 10 Reta Lila Weston Institute, UCL Queen Square Institute of Neurology, University College London, London, UK; 11 National Institute of Neurological Disorders and Stroke, NIH, Bethesda, MD, USA; 12 ASO Rapid Development Laboratory, Therapeutics Development Branch, National Center for Advancing Translational Sciences, NIH, Rockville, MD, USA; 13 Institute of Cognitive Sciences and Technologies, National Council of Research, Rome, Italy

**Keywords:** ALS, GENETICS, NEUROGENETICS, MOTOR NEURON DISEASE, C9ORF

## Abstract

**Background:**

A genetic diagnosis in Amyotrophic Lateral Sclerosis (ALS) can inform genetic counselling, prognosis and, in the light of incoming gene-targeted therapy, management. However, conventional genetic testing strategies are often costly and time-consuming.

**Objective:**

To evaluate the diagnostic yield and advantages of whole-genome sequencing (WGS) as a standard diagnostic genetic test for ALS.

**Methods:**

In this population-based cohort study, 1043 ALS patients from the Piemonte and Valle d’Aosta Register for ALS and 755 healthy individuals were screened by WGS for variants in 42 ALS-related genes and for repeated-expansions in C9orf72 and ATXN2.

**Results:**

A total of 279 ALS cases (26.9%) received a genetic diagnosis, namely 75.2% of patients with a family history of ALS and 21.5% of sporadic cases. The mutation rate among early-onset ALS patients was 43.9%, compared with 19.7% of late-onset patients. An additional 14.6% of the cohort carried a genetic factor that worsen prognosis.

**Conclusions:**

Our results suggest that, because of its high diagnostic yield and increasingly competitive costs, along with the possibility of retrospectively reassessing newly described genes, WGS should be considered as standard genetic testing for all ALS patients. Additionally, our results provide a detailed picture of the genetic basis of ALS in the general population.

## Introduction

Considerable progress has been made in unravelling the complex molecular mechanisms underlying Amyotrophic Lateral Sclerosis (ALS).^1^ Nevertheless, it is not easy to gauge the impact of these genetic advances across the ALS space. Estimates of mutation carrier rate among ALS patients seen in specialist centres may be skewed due to referral bias.^2 3^ This knowledge gap hinders efforts to develop therapies and to counsel patients effectively. For this reason, we systematically analysed a population-based cohort of ALS patients using whole-genome sequencing. Our comprehensive approach provides a snapshot of what we currently know about the genetic architecture of ALS. In the process, the current study builds on our previous work, where we showed that whole-genome sequencing is a reliable tool to assess gene burden effects.^4^

## Methods

### Patients and controls cohort

We analysed ALS patients who had been enrolled in Piemonte and Valle d'Aosta Register for ALS (PARALS). All samples have been previously analysed for mutations in the *SOD1, TARDBP*, *FUS* and *C9orf72* genes (online supplemental eMaterials). An additional 755 healthy individuals underwent whole genome sequencing and were used as control data for mutation filtering.

10.1136/jnnp-2022-328931.supp1 Supplementary data

### Sequencing and bioinformatics analysis

Whole-genome sequencing methodology and quality control filters have been detailed elsewhere^4–6^ and are described in eMaterials. We extracted variant information for 46 genes previously implicated in ALS pathogenesis (online supplemental eFigure 1). We estimated the repeat lengths of the *C9orf72* and *ATXN2* repeat expansions using ExpansionHunter—Targeted (V.0.3).^7^ For variants classification, we employed a framework based on the 2015 ACMG-AMP (American College of Medical Genetics and Genomics - Association for Molecular Pathology) guidelines.^8^ Loss-of-function and previously reported ALS variants were considered pathogenic unless present in the control cohort. The remaining rare variants (defined as minor allele frequency less than 0.0001 in the non-Finnish European population) were then classified based on computational prediction and expert review (online supplemental eMaterials) and reported if deemed to be potentially pathogenic (online supplemental eFigure 1).

### Statistical analysis

A two-tailed Fisher’s exact test was used to evaluate the genetic association between *ATXN2* CAG-repeat sizes and ALS. The burden of multiple variant carriers was assessed by a binomial test.^9^ The analyses were performed in R (V.3.6.0).

## Results

**Figure 1:**
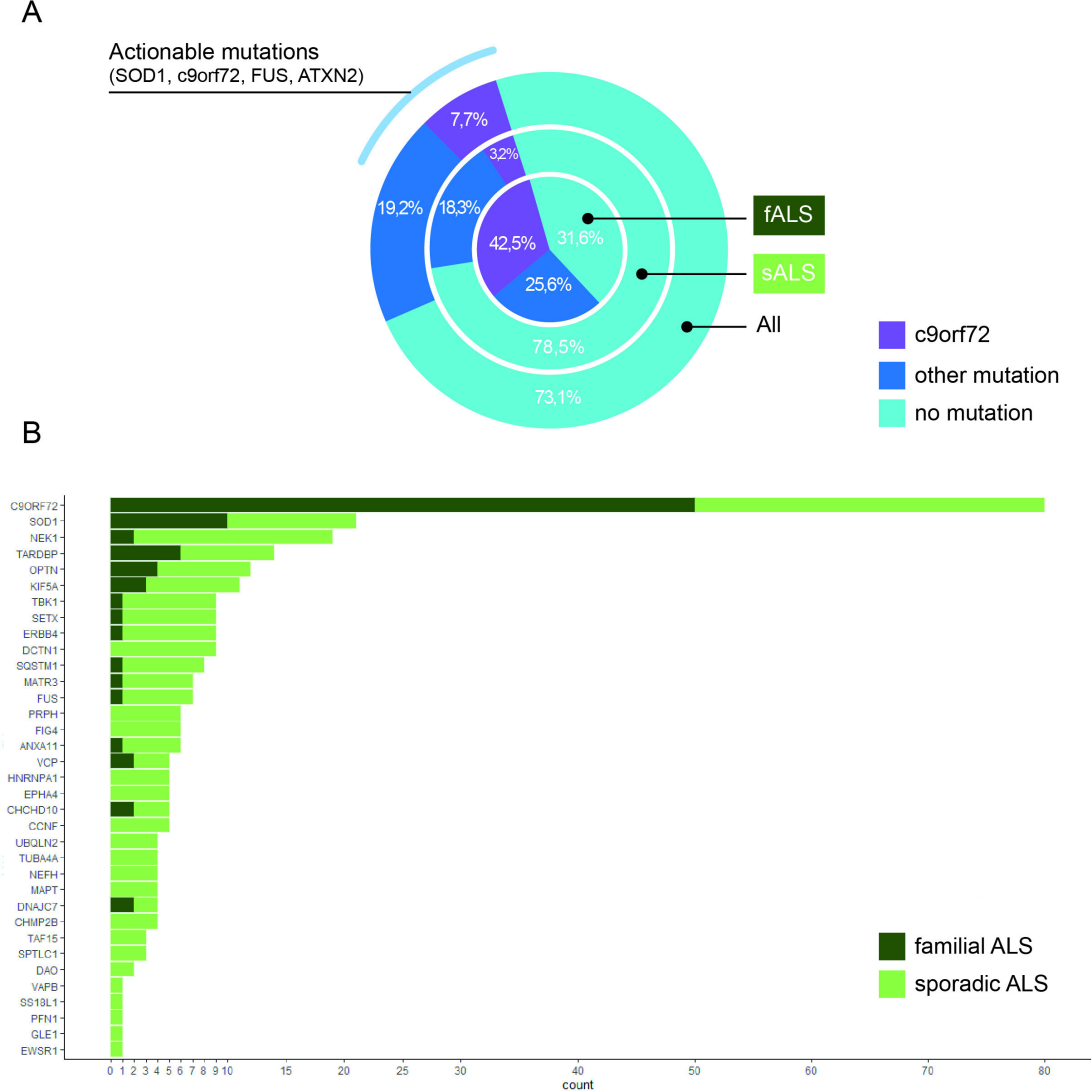
Distribution of mutated genes across our cohort. (A) The frequency of mutations in all ALS cases (out band), sporadic cases (middle band) and familial cases (inner circle). (B) The number of potentially pathogenic variants identified per gene. ALS, Amyotrophic Lateral Sclerosis

We analysed a population-based cohort of 1043 patients who had been diagnosed with ALS and enrolled in PARALS. The 1043 patients represented 71.3% of incident cases during the 2007–2016 period. We found 96 mutations known to cause ALS among 203 cases, representing 19.5% of our cohort. An additional 76 patients (7.4%) carried potentially pathogenic variants based on our classification pipeline, bringing the total number of ALS cases with disease-causing variants to approximately a quarter of ALS cases (26.9%, n=279, figure 1A andonline supplemental eTable 1). The distribution of variants across ALS genes is reported in figure 1B. The most common mutation type was C9orf72 (present in 7.7% of ALS cases), followed by SOD1 (2.0%), NEK1 (1.8%), TARDBP (1.4%) and KIF5A (0.8%). All mutations were detected by both traditional Sanger sequencing and whole-genome sequencing screening, confirming the ability of whole-genome sequencing to identify relevant mutations correctly.

### Diagnostic yield in familial and early-onset ALS cases

Subdividing our cohort, we detected disease-causing mutations in 88 (75.2%) familial ALS cases. We also observed a high mutation rate among early-onset ALS patients (age at onset <50 years, n=43, 43.9%) compared with late-onset patients (age at onset >75 years, n=41, 19.7%). Nearly half of the mutations observed in the elderly cohort were due to the *C9orf72* repeat expansion, illustrating the reduced penetrance observed with this mutation.^10^ Furthermore, 21.5% of apparently sporadic ALS patients carried a disease-causing variant (figure 1A).

### Risk factor and prognostic variants

Intermediate-length *ATXN2* CAG expansion (30–33 repeats) was the only high-risk genetic factor (defined as OR ≥2.0) identified in our cohort (OR 2.84, 95% CI 1.45 to 5.57, p=0.0023). The ATXN2 expansions were present in 41 (3.9%) patients. The prognostic *UNC13A* rs12608932 CC genotype was observed in 10.7% (n=112) of our ALS cases.

### Oligogenic cases

Our cohort’s rate of oligogenic ALS cases was 1.3% (n=13) (online supplemental eTable 2A). However, the proportion of oligogenic patients was not higher than expected (binomial p=0.98) based on the frequency of monogenic (25.6%) and non-mutated cases (73.1%) (online supplemental eTable 2B).

## Discussion

We detected a high rate of patients carrying pathogenic mutations (26.9%) in our population-based ALS cohort. This represents the largest percentage explained by genetic causes for any cohort reported to date.^2 11 12^ As we sequenced an ALS cohort from a population-based registry, our data also represent a detailed audit of what is currently known about the genetic architecture of this fatal neurodegenerative condition in the general population. Our findings reflect both our increasing knowledge of the genetic architecture of ALS and the power of whole-genome sequencing to identify these variants. For example, the C9orf72 repeat expansion accounted for nearly one-third of all cases where the mutation was known.

Our results support the use of whole-genome sequencing in ALS patients at the time of their diagnosis, irrespective of family history, age at onset or clinical phenotype.^13^ Whole-genome sequencing is a flexible tool that does not rely on a predetermined set of genes and a variant prioritisation process but can be adapted to different types of mutations as the collection of ALS-related genes evolves. The continuous improvement in variant calling and interpretation, along with our improving understanding of ALS genetics, will facilitate those endeavours. Ideally, periodic reevaluations of the whole-genome sequence data will become a feed-forward loop that enhances our knowledge with each iteration.

Our data do not support the oligogenicity theory of ALS, as it occurs very infrequently within the Italian population. The future discovery of more genes involved in ALS pathogenesis may reveal true oligogenicity. However, the existing data do not currently uphold a role for this disease mechanism.

Our primary reason for championing whole-genome sequencing as a routine test lies in the therapeutic implications for ALS patients. For example, we detected *ATXN2* intermediate trinucleotide expansion in 41 ALS subjects (3.9%) and confirmed that it increased ALS risk. This result is clinically relevant since antisense oligonucleotide therapy targeting *ATXN2* is in human trials at present.^14^ Considering *ATXN2, C9orf72*, *SOD1 and FUS*, all genes currently under study for the treatment with Antisense oligonucleotides (ASOs),^15^ at least 144 patients (13.8% of the cohort) could be candidates for gene-based therapy (figure 1A). Whole-genome sequencing allows those variants to be screened in a low-cost, rapid manner compared with traditional methodologies.

Our study is not without limitations. Above all, the lack of definite criteria for variant interpretation increases the risk of misclassifying ALS patients as wild-type or mutated. While our internal framework was reasonably robust, we do not maintain that it is definitive. Over time, the availability of updated mutation databases based on ever-larger cohort sizes and improved interpretation algorithms will enhance the analysis of these variants. Ambitious research programmes are collecting whole-genome sequencing data from millions of human genomes to pair with phenotypical and long-term clinical data.

To summarise, our population-based evaluation sheds light on the complex genetics of ALS and provides a valuable benchmark of where the field currently stands. We show that whole-genome sequencing increases diagnostic yield and facilitates the assessment of the pathogenic role of involved genes. This information will be crucial for clinical care as precision therapeutics emerge as effective treatments. In anticipation of that, we consider whole-genome sequencing as the first-tier genetic test on all ALS patients.
