# Supplementary material for: Systematic evaluation of genetic mutations in ALS: a population-based study
Source: J Neurol Neurosurg Psychiatry. 2022 Jul 27;93(11):1190–3. Online ahead of print. doi: 10.1136/jnnp-2022-328931 (PMC9606529; doi:10.1136/jnnp-2022-328931)
Supplement: Supplementary data [file jnnp-2022-328931supp001.pdf]

eMaterials

Patients and controls characteristic

Patients were included in PARALS if they met definite, probable, or probable laboratory-supported ALS diagnosis according to the revised El Escorial criteria[1]. Control samples were composed of 350 females and 405 males. The 755 controls were chosen randomly from the same population and matched to the cases by sex, age (±5 years), and geographic location. Control subjects were excluded from the study if they had a first-degree relative with a neurological or psychiatric disorder.

Standard protocol approvals and patient consents

All subjects participating in the study gave written informed consent. Ethical approval was obtained from the medical ethical review board of the A.O.U Citta della Salute e della Scienza di Torino, Italy.

Whole-genome sequencing

Whole-genome sequencing was performed at The American Genome Center at the Uniformed Services University on the Walter Reed National Military Medical Center campus in Bethesda, MD, USA. Briefly, libraries were prepared using TruSeq DNA PCR-Free High Throughput Library Prep Kit (Illumina Inc.) as per the manufacturer's instructions. Sequencing was performed on an Illumina HiSeq X10 sequencer using paired-end 150 base pair reads, and the data were processed according to Genome Analysis Toolkit's (GATK) best practices (https://software.broadinstitute.org/gatk/best-practices/). Variant quality control was performed using the GATK variant quality score method with default filters using Genome Reference Consortium Human Build 38 as the reference.

ALS-related genes

We extracted variant information from the data for the following genes: *ALS2, ANXA11, ATXN2, C21orf2, C9orf72, CCNF, CHCHD10, CHMP2B, DAO, DCTN1, DNAJC7, ERBB4, EWSR1, FIG4, FUS, GLE1, GRN, HRNPA1, HRNPA2B1, HNRNPD, KIF5A, MAPT, MATR3, NEFH, NEK1, OPTN, PFN1, PRNP, PRPH, SETX,*

SIGMAR1, SOD1, SPG11, SPTLC1, SQSTM1, SS18L1, TAF15, TARDBP, TBK1, TUBA4A, UBQLN2, VAPB, and *VCP*. Prognostic genes (*UNC13A, CAMTA1*) were also extracted.

Variant annotation

Annotation was then performed using ANNOVAR v2020-06-07 (https://annovar.openbioinformatics.org) and KGGseq v1.0 (http://pmglab.top/kggseq/) using the gnomAD database (version 2.1.1) to determine minor allele frequency (MAF) in the European-derived population. The current study was focused on coding variants due to the limitations in interpreting non-coding variants.

Expansion screening

The samples were screened for *C9orf72* expansions and the microsatellite repeat in *ATXN2* using repeat-primed PCR methodology as previously described.[2,3] A cut-off of 30 repeated expansion and the characteristic sawtooth pattern was considered pathogenic for *C9orf72*[2]. *ATXN2* CAG expansions were deemed intermediate if they were within the range of 30-33 repeats[3]. ExpansionHunter - Targeted software (version 0.3) was used to estimate repeat lengths of known, disease-causing expansions in samples undergoing whole-genome sequencing. This algorithm has been validated using experimentally confirmed samples carrying expansions[4]. The performance of whole-genome sequencing in terms of sensitivity for repeated expansion has been validated in the same ALS cohort [5,6].

Variant interpretation

We set a conservative MAF frequency threshold of <0.01% based on the epidemiology of ALS We defined Loss of Function (LoF) variants when the sequence changes were predicted to be a premature stop codon, a frameshift causing insertion/deletion (indel), or a splice-site disrupting variant located in the canonical splice sites (+1 and +2, –1 and –2) that cause the premature termination codon falling < 50–55 nucleotides upstream of the 3′ most exon–exon junctions. Loss of function variants was considered deleterious unless they exceeded the 0.01% MAF threshold. For all other type of variants, the combination of different sets of algorithms was

considered using recommended treshold [MutationTaster, Mendelian Clinically Applicable Pathogenicity (M-CAP), CADD, Variant Effect Scoring Tool (VEST3), Rare Exome Variant Ensemble Learner (REVEL), Meta-analytic Support Vector Machine (Meta-SVM)][7]. hose variants underwent an independent review by clinical and genetic ALS experts and were confirmed to be clinically reportable if agreed by consensus. When available, gene-specific information data was used in the classification

Statistical Analysis

A 2-tailed Fisher exact test was used to evaluate the genetic association between *ATXN2* CAG repeat sizes and ALS (significance set at $P < 0.05$). The burden of multiple variant carriers was assessed by a binomial test[8]. Age at disease onset and disease progression were also assessed across oligogenic and monogenic with analysis of variance (ANOVA). The analyses were performed in R (version 3.6.0).

Data availability statement

The individual-level sequence data are available on dbGaP (accession number: phs001963.v1.p1) upon motivated request by interested researchers.

eFigure 1. Study Flowchart. Simplified flowchart describing the steps taken to filter and prioritize variants in ALS genes. MAF, minor allele frequency; NFE, non-Finnish Europeans; ACMG, American College of Medical Genetic.

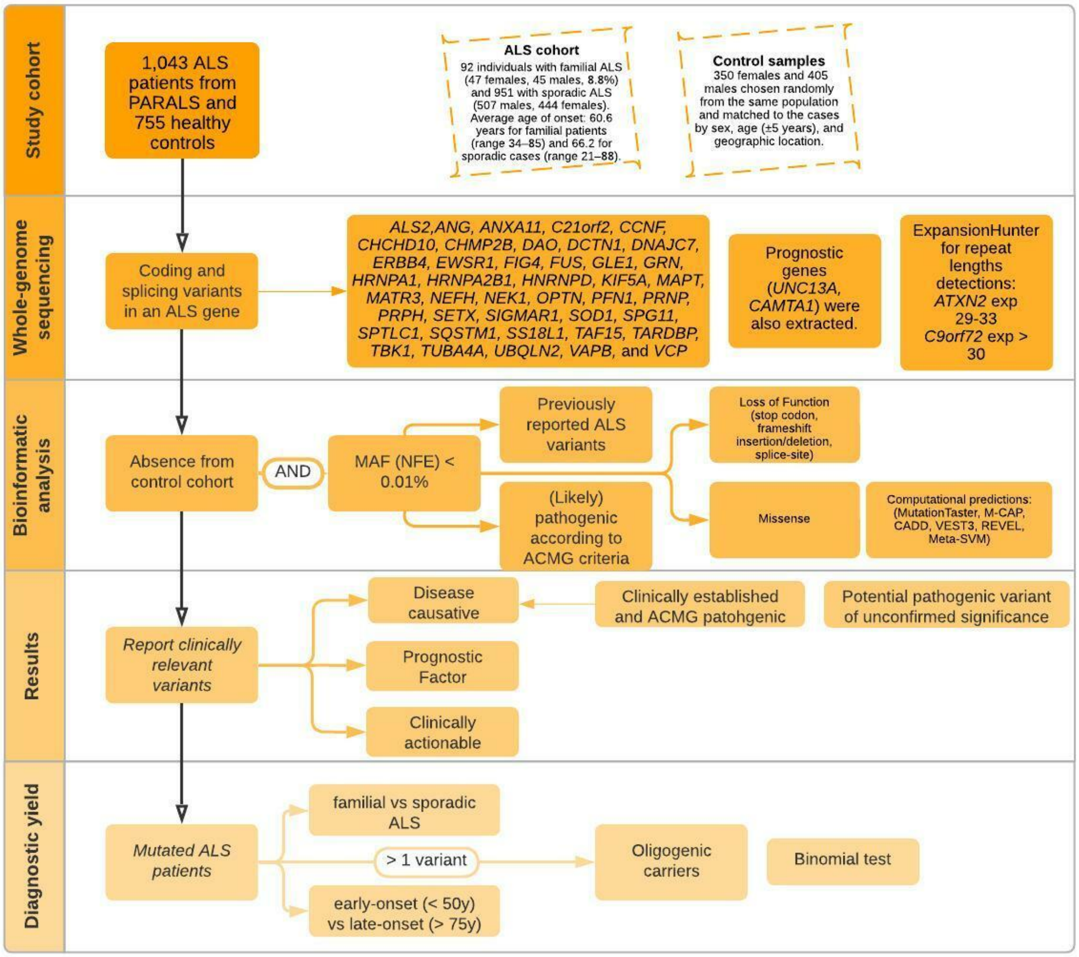

eFigure 2. Summary of identified variants.

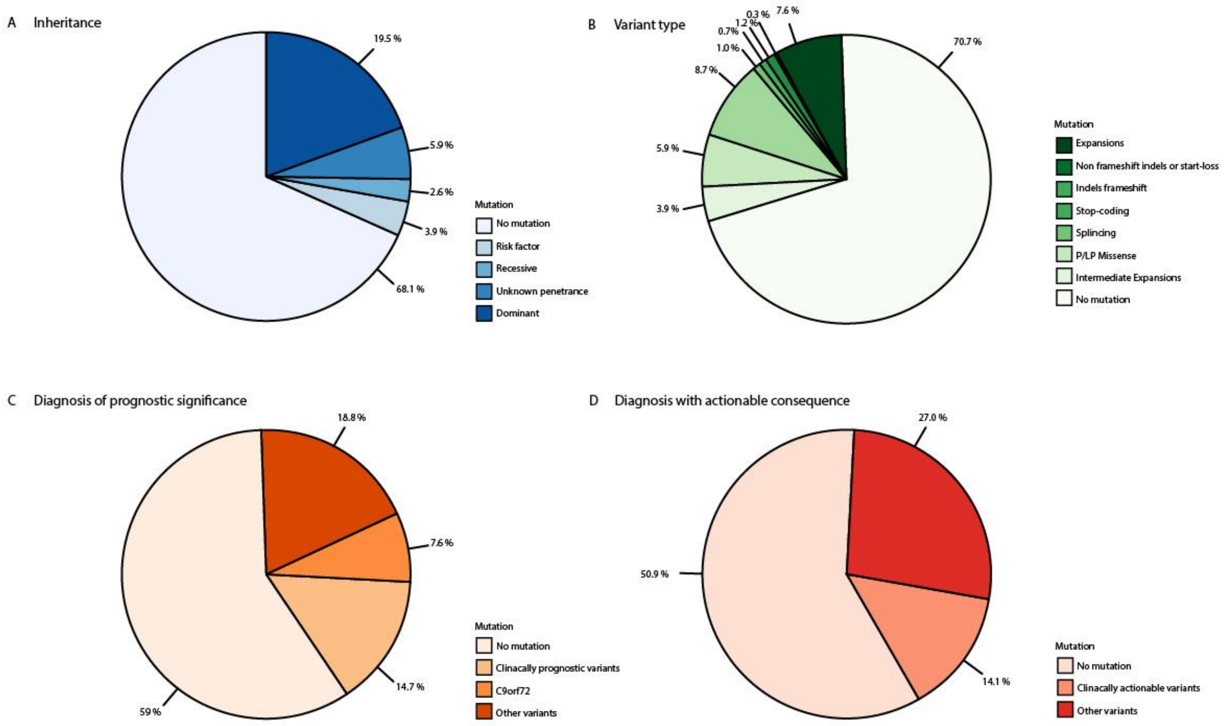

eTables

eTable 1 – Variants detected in our cohort previously reported in ALS cases.

Gene	Transcript	Nucleotide change	Amino acid change	Exon	Function
ALS2	NM_020919	c.4867C>T	p.L1623F	exon33	missense
ALS2	NM_020919	c.3836+1G>A	.	exon24 GT donor	splicing
ALS2	NM_020919	c.3797C>G	p.P1266R	exon24	missense
ALS2	NM_020919	c.3462G>T	p.Q1154H	exon21	missense
ALS2	NM_020919	c.3394C>A	p.R1132S	exon21	missense
ALS2	NM_020919	c.3206G>A	p.G1069E	exon19	missense
ALS2	NM_020919	c.2979+1G>A	.	exon17 GT donor	splicing
ALS2	NM_020919	c.2479A>T	p.T827S	exon13	missense
ALS2	NM_020919	c.1804C>T	p.R602C	exon8	missense
ALS2	NM_020919	c.1649C>T	p.P550L	exon7	missense
ALS2	NM_020919	c.1628A>G	p.D543G	exon6	missense
ALS2	NM_020919	c.1550C>G	p.A517G	exon6	missense
ALS2	NM_020919	c.1472-1G>C	.	exon6 AG acceptor	splicing
ALS2	NM_020919	c.37G>A	p.G13R	exon3	missense

ANXA11	NM_001157	c.922C>T	p.R308X	exon8	stopgain
ANXA11	NM_001157	c.102G>A	p.M34I	exon3	startloss
ANXA11	NM_001157	c.744+2C>T	.	exon7	splicing
C21ORF2	NM_001271441	c.418_420del	p.G140del	exon5	non-frameshift deletion
C21ORF2	NM_001271441.2	c.735_736insCGTGGGGAGGGAGCATGGAGCCTCACAGGGCCC	p.P245_V246insRGQGAWSLTGP	exon6	non-frameshift insertion
C21ORF2	NM_001271441.2	c.701_702insGCCTCACAGGGCCCCGTGGGGAGGGAGCATGGAGCCTCACAGGGCC	p.V235Pfs*38	exon6	frameshift insertion
CCNF	NM_001761	c.139C>T	p.L47F	exon2	missense
CCNF	NM_001761	c.316C>G	p.L106V	exon4	missense
CCNF	NM_001761	c.1043C>T	p.P348L	exon9	missense
CCNF	NM_001761	c.1132G>A	p.V378I	exon11	missense
CHCHD10	NM_213720	c.239C>T	p.P80L	exon2	missense
CHMP2B	NM_014043	c.74A>G	p.Q25R	exon2	missense
CHMP2B	NM_014043	c.85A>G	p.I29V	exon2	missense
DAO	NM_001917	c.34G>A	p.G12R	exon2	missense

DAO	NM_001917	c.992G>A	p.G331E	exon11	missense
DCTN1	NM_001190836	c.3575C>T	p.A1192V	exon28	missense
DCTN1	NM_001190836	c.3574G>A	p.A1192T	exon28	missense
DCTN1	NM_001190836	c.2588T>C	p.M863T	exon20	missense
DCTN1	NM_001190836	c.2522A>G	p.Y841C	exon20	missense
DCTN1	NM_001190836	c.2321C>G	p.P774R	exon18	missense
DCTN1	NM_001190836	c.2147C>T	p.T716M	exon17	missense
DCTN1	NM_001190836	c.1484G>C	p.R495P	exon12	missense
DCTN1	NM_001190836	c.964C>T	p.Q322*	exon8	stopgain
DCTN1	NM_001190836	c.884T>C	p.V295A	exon7	missense
DNAJC7	NM_003315	c.611G>A	p.R204Q	exon7	missense
DNAJC7	NM_003315	c.203G>A	p.R68Q	exon3	missense
EPHA4	NM_001304537	c.2060C>G	p.S687C	exon12	missense
EPHA4	NM_001304537	c.1702T>C	p.S568P	exon9	missense
EPHA4	NM_001304537	c.1058T>C	p.L353P	exon4	missense
ERBB4	NM_005235	c.3446G>T	p.G1149V	exon27	missense
ERBB4	NM_005235	c.2525G>A	p.R842Q	exon21	missense
ERBB4	NM_005235	c.1913G>A	p.W638*	exon16	stopgain
ERBB4	NM_005235	c.1912T>C	p.W638R	exon16	missense
ERBB4	NM_005235	c.1898G>C	p.C633S	exon16	missense
ERBB4	NM_005235	c.1772A>G	p.E591G	exon15	missense

ERBB4	NM_005235	c.586C>T	p.R196C	exon5	missense
ERBB4	NM_005235	c.532G>C	p.V178L	exon4	missense
ERBB4	NM_005235	c.308G>A	p.R103H	exon3	missense
EWSR1	NM_001163285	c.1447A>G	p.M483V	exon14	missense
EWSR1	NM_001163285	c.1775_1776insTTTTT	p.Q593Ffs*20	exon17	frameshift
FIG4	NM_014845	c.30C>G	p.S10R	exon1	missense
FIG4	NM_014845	c.52T>C	p.Y18H	exon1	missense
FIG4	NM_014845	c.2200G>A	p.E734K	exon20	missense
FIG4	NM_014845	c.2467C>T	p.Q823*	exon22	stopgain
FIG4	NM_014845	c.2639T>A	p.I880N	exon23	missense
FIG4	NM_014845	c.2650C>T	p.Q884*	exon23	stopgain
FUS	NM_001170634	c.785A>G	p.N262S	exon7	missense
FUS	NM_001170634	c.1480C>T	p.R494*	exon14	stopgain
FUS	NM_001170634	c.1539G>C	p.R513S	exon15	missense
FUS	NM_001170634	c.1552C>G	p.Q518E	exon15	missense
GLE1	NM_001003722	c.1771C>T	p.Q591*	exon12	stopgain
GRN	NM_002087	c.763C>A	p.L255M	exon8	missense
GRN	NM_002087	c.1562G>A	p.C521Y	exon12	missense
GRN	NM_002087	c.1595C>A	p.T532N	exon12	missense
HNRNPA1	NM_031157	c.380A>G	p.Q127R	exon4	missense

HNRNPA1	NM_031157	c.666C>G	p.F222L	exon6	missense
HNRNPA1	NM_031157	c.824G>T	p.G275V	exon8	missense
HNRNPA1	NM_031157	c.876C>G	p.N292K	exon8	missense
HNRNPA1	NM_031157	c.883G>A	p.G295R	exon8	missense
KIF5A	NM_004984	c.340C>T	p.R114*	exon4	stopgain
KIF5A	NM_004984	c.1463T>G	p.L488R	exon14	missense
KIF5A	NM_004984	c.2152C>T	p.R718W	exon19	missense
KIF5A	NM_004984	c.2263G>A	p.E755K	exon20	missense
KIF5A	NM_004984	c.2757del	p.K920Nfs*128	exon25	frameshift
MAPT	NM_016834	c.149T>C	p.I50T	exon3	missense
MAPT	NM_016834	c.736G>A	p.G246S	exon7	missense
MAPT	NM_016834	c.880T>C	p.S294P	exon9	missense
MAPT	NM_016834	c.913G>A	p.V305I	exon9	missense
MATR3	NM_018834	c.368A>G	p.D123G	exon2	missense
MATR3	NM_018834	c.2148+2T>+GGAC	.	exon14 GT donor	splicing
MATR3	NM_018834	c.2371+2T>+ATA	.	exon14 GT donor	splicing
NEFH	NM_021076	c.1036C>T	p.R346C	exon2	missense
NEFH	NM_021076	c.1723C>T	p.P575S	exon4	missense
NEFH	NM_021076	c.1783C>T	p.P595S	exon4	missense
NEFH	NM_021076	c.2461A>G	p.K821E	exon4	missense

NEK1	NM_001199399	c.3502C>T	p.H1168Y	exon32	missense
NEK1	NM_001199399	c.3451A>C	p.I1151L	exon32	missense
NEK1	NM_001199399	c.3419_3422del	p.I1140Rfs*17	exon31	frameshift
NEK1	NM_001199399	c.3373G>A	p.E1125K	exon31	missense
NEK1	NM_001199399	c.3214delA	p.T1072Lfs*20	exon30	frameshift
NEK1	NM_001199399	c.2816C>G	p.S939*	exon28	stopgain
NEK1	NM_001199399	c.2698G>T	p.D900Y	exon28	missense
NEK1	NM_001199399	c.2523_2526del	p.N841Kfs*53	exon26	frameshift
NEK1	NM_001199399	c.1226G>A	p.W409*	exon15	stopgain
NEK1	NM_001199399	c.1129_1132del	p.Q377Rfs*7	exon13	frameshift
NEK1	NM_001199399	c.781C>T	p.R261C	exon10	missense
NEK1	NM_001199399	c.577C>T	p.L193F	exon9	missense
NEK1	NM_001199399	c.464+1G>AG+	.	exon6 GT donor	splicing
NEK1	NM_001199399	c.449C>G	p.A150G	exon7	missense
NEK1	NM_001199399	c.380G>A	p.R127Q	exon6	missense
OPTN	NM_001008212	c.247C>T	p.R83C	exon4	missense
OPTN	NM_001008212	c.265C>T	p.Q89*	exon4	stopgain
OPTN	NM_001008212	c.332T>G	p.L111R	exon4	missense
OPTN	NM_001008212	c.403G>T	p.E135*	exon5	stopgain
OPTN	NM_001008212	c.644G>A	p.R215K	exon7	missense

OPTN	NM_001008212	c.917_921del	p.T307Sfs*3	exon9	frameshift
OPTN	NM_001008212	c.1499T>C	p.L500P	exon13	missense
OPTN	NM_001008212	c.1634G>A	p.R545Q	exon15	missense
OPTN	NM_001008212	c.1639C>T	p.Q547*	exon15	stopgain
OPTN	NM_001008212	c.1719G>A	p.M573I	exon15	missense
PFN1	NM_005022	c.67G>A	p.V23M	exon1	missense
PRPH	NM_006262	c.487G>T	p.D163Y	exon1	missense
PRPH	NM_006262	c.611T>A	p.V204E	exon3	missense
PRPH	NM_006262	c.996+1G>A	.	exon6 GT donor	splicing
PRPH	NM_006262	c.1024G>A	p.E342K	exon6	missense
PRPH	NM_006262.4	c.1222_1223insGCAG	p.S408Cfs*19	exon7	frameshift
PRPH	NM_006262	c.1409A>C	p.Y470S	exon9	missense
SETX	NM_015046	c.7240C>T	p.R2414*	exon25	stopgain
SETX	NM_015046	c.6848_6851del	p.T2283Kfs*32	exon22	frameshift
SETX	NM_015046	c.6685A>G	p.M2229V	exon21	missense
SETX	NM_015046	c.1427A>G	p.H476R	exon10	missense
SETX	NM_015046	c.1427A>C	p.H476P	exon10	missense
SETX	NM_015046	c.1178T>C	p.L393P	exon10	missense
SETX	NM_015046	c.638C>T	p.S213F	exon6	missense
SETX	NM_015046	c.62A>T	p.Y21F	exon3	missense

SETX	NM_015046	c.4A>G	p.S2G	exon3	missense
SOD1	NM_000454	c.16G>A	p.V6M	exon1	missense
SOD1	NM_000454	c.59A>G	p.N20S	exon1	missense
SOD1	NM_000454	c.115C>G	p.L39V	exon2	missense
SOD1	NM_000454	c.197A>G	p.N66S	exon3	missense
SOD1	NM_000454	c.217G>A	p.G73S	exon3	missense
SOD1	NM_000454	c.271G>A	p.D91N	exon4	missense
SOD1	NM_000454	c.281G>A	p.G94D	exon4	missense
SOD1	NM_000454	c.357+1G>+T	.	exon5 GT donor	splicing
SOD1	NM_000454	c.409A>T	p.K137*	exon5	stopgain
SOD1	NM_000454	c.435G>C	p.L145F	exon5	missense
SOD1	NM_000454	c.435G>T	p.L145F	exon5	missense
SOD1	NM_000454	c.442G>A	p.G148S	exon5	missense
SPAST	NM_001363875	c.455T>C	p.I152T	exon2	missense
SPAST	NM_001363875	c.806C>T	p.S269F	exon5	missense
SPAST	NM_001363875	c.1526A>G	p.D509G	exon14	missense
SPG11	NM_025137	c.6877C>T	p.R2293W	exon38	missense
SPG11	NM_025137	c.6857G>C	p.R2286P	exon38	missense
SPG11	NM_025137	c.6094T>C	p.C2032R	exon32	missense
SPG11	NM_025137	c.6010T>G	p.L2004V	exon32	missense

SPG11	NM_025137	c.5414G>A	p.R1805H	exon30	missense
SPG11	NM_025137	c.3095C>T	p.P1032L	exon17	missense
SPG11	NM_025137	c.148C>T	p.Q50*	exon1	stopgain
SQSTM1	NM_003900	c.447C>G	p.D149E	exon3	missense
SQSTM1	NM_003900	c.775G>A	p.V259M	exon6	missense
SQSTM1	NM_003900	c.775G>C	p.V259L	exon6	missense
SQSTM1	NM_003900	c.1043C>T	p.P348L	exon7	missense
SQSTM1	NM_003900	c.1084G>A	p.E362K	exon7	missense
SQSTM1	NM_003900	c.1142C>T	p.A381V	exon7	missense
SS18L1	NM_001301778	c.253A>C	p.M85L	exon7	missense
TAF15	NM_139215	c.116G>A	p.G39E	exon4	missense
TAF15	NM_139215	c.329A>G	p.Y110C	exon6	missense
TAF15	NM_139215	c.1088+1G>A	.	exon14 GT donor	splicing
TARDBP	NM_007375	c.800A>G	p.N267S	exon6	missense
TARDBP	NM_007375	c.1144G>A	p.A382T	exon6	missense
TARDBP	NM_007375	c.1169A>G	p.N390S	exon6	missense
TARDBP	NM_007375	c.1178C>T	p.S393L	exon6	missense
TBK1	NM_013254	c.254T>C	p.I85T	exon4	missense
TBK1	NM_013254	c.454G>C	p.V152L	exon5	missense
TBK1	NM_013254	c.521A>G	p.Y174C	exon5	missense

TBK1	NM_013254	c.992+1G>A	.	exon9 GT donor	splicing
TBK1	NM_013254	c.1343_1346del	p.I450Kfs*15	exon12	frameshift
TIA1	NM_022173	c.1154C>T	p.T385I	exon13	missense
TIA1	NM_022173	c.746A>C	p.K249T	exon10	missense
TIA1	NM_022173	c.95C>A	p.P32H	exon2	missense
TIA1	NM_022173	c.94C>A	p.P32T	exon2	missense
TUBA4A	NM_001278552	c.1312G>A	p.D438N	exon4	missense
TUBA4A	NM_001278552	c.1184_1190del	p.G395Afs*67	exon4	frameshift deletion
TUBA4A	NM_001278552	c.1045A>T	p.T349S	exon4	missense
TUBA4A	NM_001278552	c.148delG	p.A50Qfs*89	exon2	frameshift deletion
UBQLN2	NM_013444	c.401C>T	p.T134I	exon1	missense
UBQLN2	NM_013444	c.1172A>G	p.Y391C	exon1	missense
UBQLN2	NM_013444	c.1505G>A	p.G502D	exon1	missense
VAPB	NM_004738	c.332C>T	p.P111L	exon4	missense
VCP	NM_001354927	c.2086C>T	p.R696C	exon16	missense
VCP	NM_001354927	c.2002A>G	p.R668G	exon15	missense
VCP	NM_001354927	c.265T>C	p.Y89H	exon4	missense

eTable 2A. Oligogenic combinations in our cohort

**Mutation 1**	**Mutation 2**
C9orf72 GGGGCC exp	*CHCHD10*:c.239C>T:p.P80L
C9orf72 GGGGCC exp	*KIF5A*:c.T1463G:p.L488R
C9orf72 GGGGCC exp	*C21orf2*:c.700_701ins:p.V246 Wfs*35
C9orf72 GGGGCC exp	*NEK1*:c.G3373A:p.E1125K
C9orf72 GGGGCC exp	*OPTN*:c.G644A:p.R215K
SOD1:c.59A>G:p.N20S	*FUS*:c.1542G>C:p.R514S
SOD1:c.115C>G:p.L39V	*MATR3*:c.2148+2->GGAC
SOD1:c.271G>A:p.D91N	*CHCHD10*:c.239C>T:p.P80L
SOD1:c.435G>C:p.L145F	*OPTN*:c.403G>T:p.E135*
SOD1:c.435G>C:p.L145F	*ERBB4*:c.308G>A:p.R103H
TARDBP:c.1144G>A:p.A382T	*UBQLN2:*c.1612G>C:p.V538L
DCTN1:c.C673T:p.Q225*	*MATR3*:c.2148+2->GGAC
ANXA11:c.C922T:p.R308X	*ERBB4*:c.A1772G:p.E591G

eTable 2B. Analysis of oligogenic frequency and effect of multiple variants on age at disease onset in our cohort

	**Oligogenic cases**	**Monogenic cases**	**Binomial p-value**	**ANOVA p-value**
**Cases (% cohort)**	13 (1.3%)	266 (25.6%)	0.98	-
**Age at onset (SD)**	59.9 (11.9)	63.9 (11.2)	-	0.1310

Supplementary References

1. Brooks BR, Miller RG, Swash M, Munsat TL. El Escorial revisited: Revised criteria for the diagnosis of amyotrophic lateral sclerosis. *Amyotrophic Lateral Sclerosis and Other Motor Neuron Disorders*. 2000;1(5). doi:10.1080/146608200300079536

2. Renton AE, Majounie E, Waite A, et al. A Hexanucleotide Repeat Expansion in C9ORF72 Is the Cause of Chromosome 9p21-Linked ALS-FTD. *Neuron*. 2011;72(2). doi:10.1016/j.neuron.2011.09.010

3. Van Damme P, Veldink JH, van Blitterswijk M, et al. Expanded ATXN2 CAG repeat size in ALS identifies genetic overlap between ALS and SCA2. *Neurology*. 2011;76(24):2066-2072. doi:10.1212/WNL.0b013e31821f445b

4. Dolzhenko E, van Vugt JJFA, Shaw RJ, et al. Detection of long repeat expansions from PCR-free whole-genome sequence data. *Genome Research*. 2017;27(11). doi:10.1101/gr.225672.117

5. Grassano M, Calvo A, Moglia C, et al. Mutational analysis of known ALS genes in an Italian population-based cohort. *Neurology*. Published online November 2020. doi:10.1212/WNL.0000000000011209

6. Dewan R, Chia R, Ding J, et al. Pathogenic Huntingtin Repeat Expansions in Patients with Frontotemporal Dementia and Amyotrophic Lateral Sclerosis. *Neuron*. 2021;109(3):448-460.e4. doi:10.1016/j.neuron.2020.11.005

7. Anderson D, Lassmann T. A phenotype centric benchmark of variant prioritisation tools. *npj Genom Med*. 2018;3(1):5. doi:10.1038/s41525-018-0044-9

8. Morgan S, Shatunov A, Sproviero W, et al. A comprehensive analysis of rare genetic variation in amyotrophic lateral sclerosis in the UK. *Brain*. 2017;140(6):1611-1618. doi:10.1093/brain/awx082
